# BRCA1 Protein Expression Level and CD44^+^Phenotype in Breast Cancer Patients

**Published:** 2011-09-23

**Authors:** Zahra Madjd, Adel Karimi, Saadat Molanae, Mohsen Asadi-Lari

**Affiliations:** 1. Department of Pathology, Tehran University of Medical Sciences, Tehran, Iran; 2. Oncopathology Research Centre, Tehran University of Medical Sciences, Tehran, Iran; 3. Departmentof Pathology, Milad Hospital, Tehran, Iran

**Keywords:** Breast Cancer, Cancer Stem Cells, *BRCA1*, Immunohistochemistry

## Abstract

**Objective:**

CD44^+^/CD24^-/low^ breast cancer cells have tumour-initiating properties with stem cell-like features. Breast cancer gene 1 *(BRCA1)* is a tumour suppressor gene that plays a crucial role in DNA repair and maintenance of chromosome stability. The clinicopatho- logical features of breast cancer in *BRCA1* mutation carriers suggest that *BRCA1* may function as a stem-cell regulator.

**Materials and Methods:**

In the present experimental study we examined the expression and localization of the *BRCA1* protein and investigated the prognostic value as well as its relationship with the putative cancer stem cell (CSC) marker (CD44) in 156 tumour samples from a well-characterized series of unselected breast carcinomas using immunohistochemistry. Statistical analysis of the data was performed using SPSS software version 16 (Chicago, IL, USA).

**Results:**

In breast tumours, the loss of nuclear expression was detected in 23 cases (15%), whereas cytoplasmic expression of *BRCA1* was observed in 133 breast carcinomas (85%). Altered *BRCA1* expression was significantly associated with high grade and poor prognosis breast tumours (p=0.006). We further established an inverse significant correlation between *BRCA1* expression levels and CD44^+^ cancer cell phenotype (p=0.02).

**Conclusion:**

Loss of BRCA1 expression is a marker of tumour aggressiveness and correlates with CD44^+^ tumour cell phenotype. Taken together, the present study supports the idea that the loss of *BRCA1* results in persistent errors in DNA replication in breast stem cells and provides targets for additional carcinogenic events.

## Introduction

 Breast cancer is the most commonly occurring cancer and the second leading cause of cancer death among women in Western countries(1). About 5%-10% of all breast cancers can be attributed to highly penetrative germline mutations such as breast cancer gene 1 (BRCA1) and BRCA2 (2). *BRCA1* is located on chromosome 17q21 (3). Up to age 40 , women with *BRCA1* are estimated to have a 20-fold greater risk of breast cancer compared to the general population and 60 %-85 % lifetime risk of breast cancer (4). A recent study of the Iranian population showed that screening for *BRCA1* and *BRCA2* mutations in high risk populations has a strong influence on health-care (5). Based on the cancer stem cell (CSC) hypothesis, cancers arise from stem or progenitor cells through dysregulation of the self-renewal process. This generates tumours that are driven by a cellular subcomponent that maintains CSC properties (6). CSCs have been identified in breast carcinomas (7) as well as hematopoietic malignancies and many solid tumours (8-10).

 It has been proposed that genes related to hereditary cancers, such as *BRCA1*, participate in regulating stem cell fate(11, 12). Mouse models have established an important role for BRCA1 in mammary gland development, emphasising the relationship between development and cancer (13). Human studies have also proposed that *BRCA1* plays a role in the determination of the architecture and function of the adult breast (14). *BRCA1* has a key role in DNA repair and functions in cell cycle control and maintenance of genetic stability (15). Loss of these functions by *BRCA1* may result in the accumulation of genetically unstable breast stem cells, which provide targets for additional carcinogenic procedures (12). Wright and colleagues have shown cellular heterogenicity of CSCs by using a mouse knockout model of *BRCA1*. A subpopulation from one tumour with stem cell properties was CD44^+^/ CD24^-^, whereas cells that have been derived from another tumour contained a CSC population with CD133 expression. This study has shown no overlap between two cell populations, suggesting that breast tumours may exhibit inter-tumour cell heterogenicity in *BRCA1* tumours which may result from different cells of origin. Although there was no overlap in CD44^+^/CD24^-^ and CD133 subpopulations, both CSC populations over expressed stem cell genes Oct4, Notch1, Sox1, and ALDH1 and both displayed resistance to chemotherapeutic agents (16, 17).

 Despite this heterogenicity, breast CSCs possess common characteristics based on their surface makers that can be used for their identification (18). The subpopulation with CD44^+^/CD24^-^ phenotype has been previously described as CSC in breast tumours (7). Some molecules, such as adhesion molecule CD44, can be empirically selected as a prospective marker of CSCs. Although much is known about the molecular genetics of *BRCA1* in breast cancer, its association with CSC markers has not been studied in detail.

 In the present study, to evaluate the pattern of expression and prognostic significance of BRCA1 in breast tumours, we analysed the expression of the *BRCA1* protein in a series of unselected breast cancer cases by immunohistochemistry. The pattern of *BRCA*1 expression was then correlated with expression of CD44 (CD44^+^ cancer stem cell phenotype) in the same collection of breast tumours. To our knowledge, no immunohistochemical data was available regarding the relationship between CSC marker CD44 and BRCA1 in an unselected series (either family or sporadic) of breast carcinomas.

## Materials and Methods

### Patients and tumour samples

 A total of 156 tumour samples from breast cancer patients who underwent breast surgery or biopsy between 2006 and 2007 at Milad Hospital, a major public referral centre in Tehran, Iran, were included in this study. Surgical specimens were obtained before systemic treatment and paraffin embedding was performed within the framework of diagnostic procedures. The following data were sought from the patients' medical records as part of the study: age, tumour size, vascular invasion (19), tumour grade (20), stage/lymph node status, and tumour type (21). The selection of these patients was not on the basis of age at diagnosis or family history of breast/ovarian cancer. Patients'data were kept fully anonymous. This collection of primary operable breast carcinomas was previously used to study CD44 and Bcl_2_ proteins (22), which were compared with the results obtained from BRCA1. This study was approved by the Tehran University of Medical Sciences (TUMS) Research Ethics Committee. The patients filled the informed consent forms at the beginning of study.

### Immunohistochemistry

 The labelled strepatavidin biotin (LSAB) method was used on formalin-fixed paraffin embedded (FFPE) tissue sections (4 µm) as previously described (22). Briefly, tissue slides were deparaffinised with xylene and then rehydrated in descending concentrations of alcohol. Endogenous peroxidase activity was blocked by incubation in a 0.3% hydrogen peroxide/methanol buffer for 15 minutes. Antigens were retrieved by autoclaving for 10 minutes in sodium citrate buffer (pH=6.0). The slides were incubated with the *BRCA1* monoclonal antibody (clone MxH GLK2, DakoCytomation, Glostrup, Denmark) at an optimal dilution of 1:40 for 1 hour at room temperature. After washing with tris buffered saline (TBS) , tissues were incubated in biotinylated goat anti-mouse/rabbit IgG (DakoCytomation, Glostrup, Denmark) for 30 minutes followed by horseradish peroxidase (HRP) labelled streptavidin complex (DakoCytomation) for a further 1 hour at room temperature with the addition of 3, 3'-diaminobenzidine (DAB, Dako) as a chromogen to achieve visualization of the antigen. Finally, all sections were lightly counterstained with haematoxylin (DakoCytomation), dehydrated in alcohol, cleared in xylene and mounted for examination. Normal breast tissues adjacent to the tumours and infiltrating leukocytes were used as positive controls. The primary antibody was omitted from the negative control slides in every experiment. CD44 expression data were available from the previous published work that used monoclonal anti-CD44 (clone DF185, Novocastra), as were data concerning Bcl_2_ expression (clone 124, Dako) in the same series of patients (22).

### Evaluation of immunohistochemical staining

 Staining assessment was based on a semi-quantitative scoring system that relies on the subjective assessment of multiple independent observers, or one observer on two separate occasions blinded to patient outcomes and clinicopathological data. In the present study, the staining of tissue sections was interpreted by two observers (Zahra Madjd and Adel Karimi) on two separate occasions and a consensus was achieved between the two scorings.

Initially, the slides were scanned at 10x magnification to obtain a general impression of the overall distribution of the tumour cells. The proportion of positive cells was then assessed semi-quantitatively at higher magnifications and the final scores were given. Three scoring methods were employed to assess the level of *BRCA1* expression in breast tumour sections: First, the intensity of the immunostaining: a score index of 0, 1, 2 and 3 corresponded to negative, weak, moderate, and strong staining (23). Secondly, the percentage of stained cells was estimated subjectively, then classified into four groups: 1 (<25% positive cells), 2 (25- 50% positive cells), 3 (51-75% positive cells), and 4 (>75% positive cells). Finally, the modified histochemical score (H-score) (24) was obtained by multiplying the intensity of staining and percentage of positive cells and a final score of 0 to 300 was given. The expected pattern of *BRCA1* expression was nuclear, cytoplasmic, or combined nuclear and cytoplasmic staining. The cut-off value for positive nuclear expression was the median of expression (H-score=160). A further classification was therefore performed for positive nuclear expression based on values below the cut-off (median) to demonstrate reduced expression, and above the cut-off to demonstrate strong expression (23).

### Statistical analysis

 Statistical analysis of the data was performed using SPSS software version 16 (Chicago, IL). The significance of the associations between *BRCA1* expression and clinicopathological parameters were analysed using Pearson's chi-square and Pearson's R tests. To obtain effect sizes and to look at the independence of effects, *BRCA1* nuclear expression was recategorized into two groups as high(strong) and low (loss/reduced,) and effects of clinicopathological parameters were assessed using multiple logistic regression to give adjusted odds ratios and 95% confidence intervals. The Wilcoxon signed-rank test was used to compare the H-scores of CD44 and *BRCA1*. P-values less than 0.05 were considered statistically significant.

## Results

### Study population

 Of these female patients, 23% were younger than 40 years old, while 77% were over 40. At the time of diagnosis, patients had a median age of 47 years (range: 25–82 years). Of the 156 breast carcinoma cases with available data, the majority of tumours were grade 3 (47%) or grade 2 (40%), and only 13% of the cases were grade 1. The most common histological type was invasive ductal carcinoma that comprising 89% of the cases. Tumour size was categorised in two main groups based on TNM classification [primary tumour (T), regional lymph nodes (N) and distant metastasis (M)] of human breast cancers: group 1 tumours were 2.0 cm or less in greatest dimension (T1) and comprised 30% of the tumours. Group 2 tumours were more than 2.0 cm in greatest dimension (T2, T3 and T4) and included 70% of the tumours. Of the patients with known lymph node status, 64% were lymph node positive (one or two auxiliary nodes involved), and 36% were node negative. Vascular invasion was seen in 37% of the tumours. Patients and tumour characteristics, also nuclear *BRCA1* immunohistochemical reaction results are summarized in Table 1.

### BRCA1 expression in breast carcinomas

**Fig 1:**
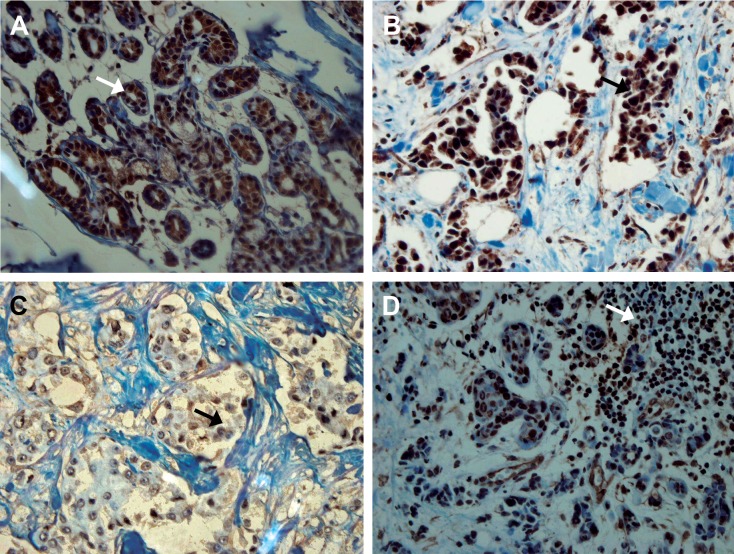
Nuclear expression of *BRCA1*. A. Strong nuclear staining in normal breast duct adjacent to tumour. B. A case of invasive ductal carcinoma showing strong positive *BRCA1* nuclear expression. C. A case of invasive ductal carcinoma with reduced *BRCA1* nuclear expression. D. Infiltrating lymphocytes showing strong positive *BRCA1* expression. Original magnification: (A, B, D) ×20, (C) ×40.

**Fig 2:**
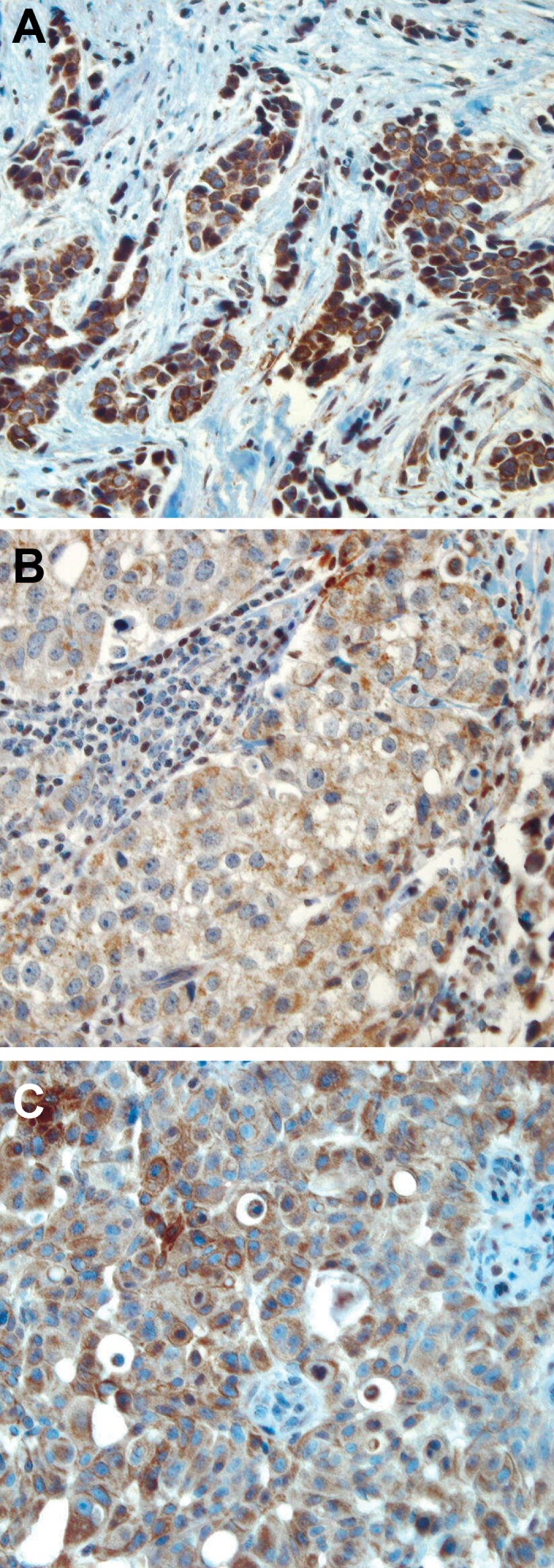
Cytoplasmic expression of *BRCA1* protein. (A) Strong, (B) moderate, and (C)weak cytoplasmic expression of *BRCA1* protein observed in invasive breast carcinomas original magnification: (A) ×20, (B, C) ×40.

**Fig 3:**
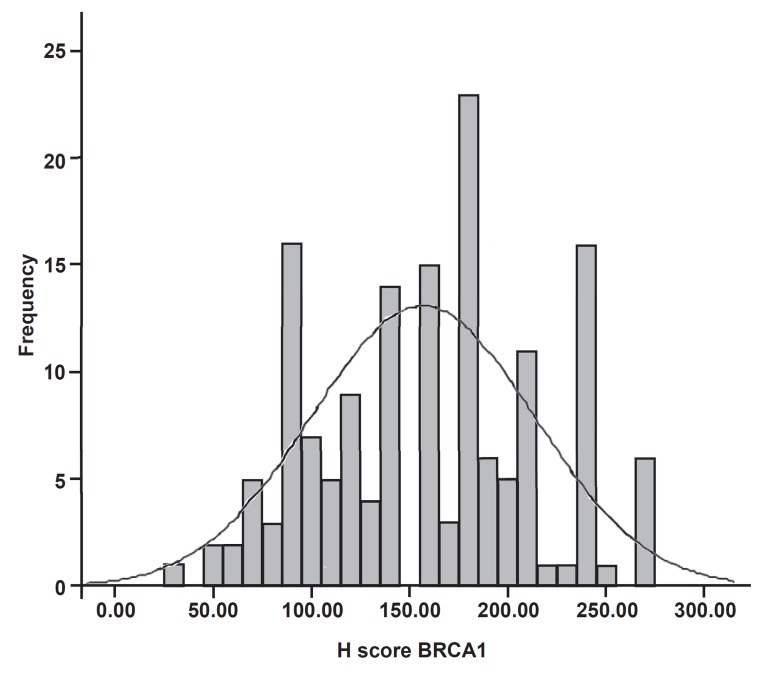
Histogram showing distribution of BRCA1 H-score values

 Adjacent normal breast tissue, wherever present, stained strongly positive for *BRCA1*. The staining of normal ducts was localized to the nuclei of the cells and no cytoplasmic or membranous staining was observed (Fig 1A). In contrast, the immunohistochemical expression of *BRCA1* within the breast tumours was broadly heterogeneous and the intensity of staining was lower than in the normal breast. The staining pattern of expression was either nuclear (Fig 1B, C),cytoplasmic, or both nuclear and cytoplasmic (Fig 2A,B,C). For H-score determination, the cut-off value (median) was calculated to define groups that showed both strong (H-score>160) and reduced (H-score<160) expressions of *BRCA*1. The distribution of *BRCA1* H-score (0-300) is showed in figure 3. Of 156 breast tumours that stained with *BRCA1*, complete loss of nuclear expression was detected in 23 (15%) cases. Reduced expression was seen in 65 (42%), whereas strong staining was observed in 68 (43%) cases.

 A total of 85%(133/156) of the tumours showed cytoplasmic pattern that correlated with nuclear pattern, while membranous staining was detected in only 10% (16 /156) of tumours. No *BRCA1* protein staining was observed in the stroma of malignant breast tissues, whereas infiltrating lymphocytes showed positivity for *BRCA1* (Fig 1D). Both normal breast tissue adjacent to the tumours and infiltrating lymphocytes within the tumours that showed strong and uniform staining of *BRCA1*, were used as internal positive controls.

### Association of nuclear BRCA1 expression and clinicopathological parameters

Altered *BRCA1* expression (loss or reduced nuclear expression) was more often seen in early onset

**Table 1:** Association between nuclear expression of *BRCA1* with clinicopathological parameters in breast carcinoma


Patients and tumour characteristics	Loss No.(%)	Reduced No.(%)	Strong No.(%)	Total No.(%)	
Age (years)						
≤40	4 (19)	12 (57)	5 (24)	21 (23)	0.04	
>40	7 (10)	30 (42)	34 (48)	71 (77)		
	
Histological grade						
Grade 1	0 (0)	4 (33)	8 (67)	12 (13)		
Grade 2	3 (8)	17 (44)	19 (48)	39 (40)		
Grade 3	9 (20)	22 (48)	15 (32)	46 (47)	0.006	
	
Lymph node metastasis						
Negative	3 (12)	11 (42)	12 (46)	26 (36)		
Positive	7 (15)	23 (49)	17 (36)	47 (64)	0.43	
	
Vascular invasion						
Negative	10 (18)	25 (45)	21 (37)	56 (63)		
Positive	6 (18)	13 (39)	14 (43)	33 (37)	0.77	

Tumour size (cm)						
≤ 2	2 (7)	14 (48)	13 (45)	29 (30)		
> 2	11(16)	32 (46)	26 (38)	69 (70)	0.28	

breast cancer patients (≤40 years), whereas patients over the age of 40 showed strong *BRCA1* expression at the nuclear site (p=0.04). Absent or reduced *BRCA1* nuclear expression was significantly associated with high-grade breast tumours (p=0.006).

 Altered *BRCA1* expression was more frequent in invasive ductal carcinoma and less frequent in other tumour types, including intraductal carcinoma and lobular carcinomas (p=0.02; Table 1). In multiple logistic regression, *BRCA1* nuclear expression was reclassified in 2 groups as high (strong) and low (absent or reduced). The odds ratio for high nuclear expression of *BRCA1* in tumours with a poor histological grade compared to well-differentiated tumours was 0.24 (95 %CI=0.065-0.933; Table 2).

**Table 2:** Logistic regression analysis of *BRCA1* nuclear expression recategorized into two groups, high (strong) and low (loss/reduced)


Prognostic factors	Odds ratio (95% CI)	Test for trend (linear by linear)
Age (years)			
≤ 40 years	1		
> 40 years	2.94 (0.97-8.89)	0.04	
Histological grade			
Grade 1	1		
Grade 2	0.47 (0.12-1.84)		
Grade 3	0.24 (0.06-0.93)	0.007	
Lymph node meastasis			
Negative	1		
Positive	0.66 (0.25-1.75)	0.27	
Vascular invasion			
Negative	1		
Positive	1.20 (0.46-3.11)	0.80	
Tumour size			
≤2	1		
>2	0.87 (0.36-2.08)	0.46	
Tumour type			
Invasive ductal carcinoma	1		
Other tumour types (intraductal carcinomas and invasive lobular carcinomas)	1.63 (0.40-6.58)	0.08	


 However, no association was detected between the nuclear expression of *BRCA1* in these breast carcinomas and other prognostic factors including lymph node metastasis (p=0.43), absence or presence of vascular invasion (p=0.77) or tumour size (p=0.28; Table 1). No significant correlation was found between cytoplasmic expression of *BRCA1* and any prognostic factor including tumour grade (p=0.58), lymph node metastasis (p=0.27), tumour size (p=0.87), tumour type (p=0.19), vascular invasion (p=0.07), and age at the time of diagnosis (p=0.38).

**Table 3:** Comparison of H-score between *BRCA1* and CD44


	N	Mean	Std. deviation	P-value	Minimum	Maximum	Percentiles 25^th^	50^th^(Median)	75^th^	
BRCA1 H-score	156	158.6538	56.92198	0.001	30.00	270.00	110.0	160.0	200.0	
CD44 H-score	131	84.6565	85.62259		0.00	300.00	10.0	60.0	140.0	


### Comparison of BRCA1 and CD44 expression

 To evaluate the relationship between *BRCA1* expression and the breast CSC marker CD44, we correlated the cytoplasmic and nuclear expressions of *BRCA1* with the level of expression of CD44 obtained from our previous study in the same series of breast tumours (22).

 There was a clear inverse correlation between the intensity of CD44 expression and *BRCA1* nuclear expression in this series of breast cancers, indicating that higher expression of *BRCA1* was associated with lower CD44 expression (p=0.04). A significant association was also found between the percentage of CD44^+^ cells and *BRCA1* defective tumours (p<0.001).

 Further analysis conducted for the H-score determination of CD44, which was categorised into 2 groups based on the values below and above the median (cut-off=60), demonstrated a significant inverse relationship between the CD44 H-score and *BRCA1* H-score (p=0.04). Additional non- parametric analysis (Wilcoxon signed-rank), using the raw data on both CD44 and *BRCA1* H-scores revealed a contradictory correlation between these two markers (z=-6.18, p<0.001; Table 3).

## Discussion

*BRCA1* is an important susceptibility gene for breast cancer that increases the lifetime risk of breast cancer, particularly in the pre-menopausal age group. Although it is well established that mutated *BRCA1* is associated with the development of breast and ovarian cancers, the molecular mechanisms of this tissue-specific carcinogenesis are still unclear. Recent evidence supports the hypothesis that *BRCA1* is involved in breast cancer functions as a breast stem cell regulator (11, 12, 25). Multiple studies have indicated a CD44^+^/CD24^-^ phenotype for breast CSCs (7, 26). Wright et al., using 16 cell lines from distinct *BRCA1* deficient mouse mammary tumours , found that these tumours harbor heterogeneous CSC populations and that CD44^+^/ CD24^-^ cells represent a population that correlates with human breast CSCs (18).

 In the present study, we examined the expression tic value in 156 unselected breast tumour samples (either family or sporadic breast cancer). To examine the relationship between *BRCA1* and CD44, the pattern of expression of *BRCA1* was also associated with the expression of CSC marker CD44 (22) in this collection of breast tumours. There was a strong homogeneous nuclear expression of *BRCA1* in adjacent normal breast tissue, whereas malignant tissues were broadly heterogeneous and often less intense than normal tissues. Fifty seven percent of breast tumours revealed absent (15% ) or reduced (42% ) nuclear expression of BRCA1,while 43% of the cases showed strong nuclear expression. Cytoplasmic expression was seen in 85% of breast carcinomas that were associated with a nuclear pattern in the majority of the cases, with the exception of 44 cases. Earlier publications have also shown a range of expression and subcellular localization of *BRCA1* from nuclear to cytoplasmic in breast tumour cells and nuclear staining in normal tissues (27-31). Rakha et al. in a large and well-characterized series of breast carcinomas using tissue microarray and immunohistochemistry, found a strong uniform nuclear expression of *BRCA1* in normal breast tissue while malignant tis- sues only showed an altered expression of *BRCA1* (absent or reduced nuclear expression or positive cytoplasmic expression) (23).

 We observed an altered expression of *BRCA1* more frequently in early onset breast cancer patients. Absent or reduced *BRCA1* expression was seen more in high-grade breast carcinoma compared to better-differentiated tumours. These findings were in accordance with prior studies in breast cancer, which demonstrated that mutation positive tumours or altered *BRCA1* expression was associated with poor prognosis parameters (11, 23, 32, 33).

 No association was detected between nuclear expression of *BRCA1* and other prognostic factors, including lymph node metastasis, the absence or presence of vascular invasion, or tumour size in this series of breast carcinomas. However, Rakha et al. noted that reduced *BRCA1* was associated with an advanced lymph node (LN) stage, large tumour size, and definite vascular invasion (23). The lack of association between expression of *BRCA1* and these tumour characteristics warrants further investigation with a larger number of samples.

 In contrast to nuclear expression, we found no significant correlation between cytoplasmic staining of *BRCA1* and prognostic parameters. However, previous studies that evaluated the expression of *BRCA1* and breast cancer prognosis have demonstrated various results. Taylor et al. reported both nuclear and cytoplasmic expression in the majority of normal breast ducts, and no association between cytoplasmic staining and clinical characteristics(32), while Fraser et al. (34) showed no correlation with outcome or tumour parameters.

 We also investigated the correlation between cytoplasmic/nuclear expression of *BRCA1* protein with the level of expression of CD44, as a marker of breast CSCs (22).

 Our results showed a significant correlation between *BRCA1* expression levels and CD44^+^ status in terms of intensity and percentage of positive cells, in addition to the H-scores of CD44 and *BRCA1*. These scores demonstrated that a lower expression of *BRCA1* was more often seen in tumours with higher expressions of CD44.

 Several studies have suggested a link between BRCA1 deficiency and breast CSCs. Foulkes proposed that *BRCA1* functioned as a breast stem cell regulator and predicted that breast CSCs were more likely than non-stem cells to express low levels of *BRCA1* protein (11). The regulatory role of *BRCA1* in human breast stem/progenitor cell fate has been established in previous studies (12), suggesting that a loss of *BRCA1* may lead to an accretion of unstable stem cells. Other studies have also demonstrated an association between *BRCA1* hereditary breast cancer and the presence of CD44^+^/CD24^-^ cells (35, 36), whereas our study points to the relationship between *BRCA1* and CSC marker CD44 in unselected breast cancer patients rather than only in hereditary breast cancer patients.

 This study was restricted to limited sample size and therefore warrants further investigation with a larger number of samples applying tissue microarray to detect breast CSCs, where either well established various markers such as CD44/ CD24 could be identified by a double staining method or newly introduced universal CSC markers such as ALDH1 could be used.

## Conclusion

 Further to previous studies, we have found a significant inverse relationship between the two phenotypes under investigation, *BRCA1* and CD44^+^, indicating that these tumour cells may be a subpopulation of tumourigenic cells. Loss of *BRCA1* expression is a marker of tumour aggressiveness, potentially linked to *BRCA1* status and a CSC phenotype in primary breast cancer. Breast CSCs are more likely to have low levels of *BRCA*1 expression than non-stem cells. Our results support the idea that the loss of *BRCA1* expression may result in an accumulation of genetically unstable breast stem cells, providing targets for more carcinogenic events.
